# Enhanced Electromagnetic Interference Shielding Properties of Immiscible Polyblends with Selective Localization of Reduced Graphene Oxide Networks

**DOI:** 10.3390/polym14050967

**Published:** 2022-02-28

**Authors:** Yiming Meng, Sushant Sharma, Jin Suk Chung, Wenjun Gan, Seung Hyun Hur, Won Mook Choi

**Affiliations:** 1 School of Chemical Engineering, University of Ulsan, Daehakro 93, Namgu, Ulsan 44610, Korea; 15026557980@163.com (Y.M.); sushant.nplindia@gmail.com (S.S.); shhur@mail.ulsan.ac.kr (S.H.H.); wmchoi98@ulsan.ac.kr (W.M.C.); 2 Department of Macromolecular Materials and Engineering, College of Chemistry and Chemical Engineering, Shanghai University of Engineering Science, Longteng Road 333, Shanghai 201620, China

**Keywords:** DGEBA/PEI/RGO nanocomposites, selective localization, double percolation, EMI shielding

## Abstract

Herein, an effective technique of curing reaction-induced phase separation (CRIPS) was used to construct a reduced graphene oxide (RGO) network in the immiscible diglycidyl ether of the bisphenol A/polyetherimide (DGEBA/PEI) polyblend system. The unique chemical reduction of RGO facilitated the reduction of oxygenated groups and simultaneously appended amino groups that stimulate the curing process. The selective interfacial localization of RGO was predicted numerically by the harmonic and geometric mean technique and further confirmed by field emission transmission electron microscopy (FETEM) analysis. Due to interfacial localization, the electrical conductivity was increased to 366 S/m with 3 wt.% RGO reinforcement. The thermomechanical properties of nanocomposites were determined by dynamic mechanical analysis (DMA). The storage modulus of 3 wt.% RGO-reinforced polyblend exhibited an improvement of ~15%, and glass transition temperature (*T_g_*) was 10.1 °C higher over neat DGEBA. Furthermore, the total shielding effectiveness (SE_T_) was increased to 25.8 dB in the X-band region, with only 3 wt.% RGO, which represents ~99.9% shielding efficiency. These phase separation-controlled nanocomposites with selective localization of electrically conductive nanofiller at a low concentration will extend the applicability of polyblends to multifunctional structural nanocomposite applications.

## 1. Introduction

Multifunctional polymer nanocomposites are progressively replacing metallic parts because of their superior properties, such as low density, excellent mechanical properties, low cost, and chemical stability, etc. However, commercial polymers are electrically insulating in nature and usually require highly conducting fillers that are included in the category of conducting nanocomposites. The introduction of an electrically conducting nanofiller at high concentration is one of the most efficient approaches for developing conducting polymer composites (CPCs). Therefore, many efforts have aimed to develop this strategy to achieve desired electrical conductivity. These include the introduction of electrically conducting fillers such as carbon nanotubes (CNT) [1], carbon dots [2], graphene [3], and their derivatives for CPCs [4].

With the rapid development of communications and internet technologies, electromagnetic interference (EMI) has emerged as a serious threat to the stable functioning of electronic devices [5,6,7,8]. The application of CPCs with nanofillers is one of the solutions. The addition of electrically conducting nanofillers increases the shielding properties of material via various mechanisms, such as conductive losses, destructive interference, hysteresis losses, and heating losses, etc. Zhang et al. prepared laminar structures of polyethylene oxide (PEO)/CNT (as shielding layer) and cellulose (as a supporting substrate) with high loading of nanofiller CNT at 40 wt.%, which yielded shielding effectiveness of 30–40 dB in the X band [9]. Zeng et al. successfully prepared a reinforced waterborne polyurethane (WPU) film with 61.5 wt.% of multi-walled carbon nanotubes (MWCNT), and its shielding effectiveness reached 35 dB [10]. Liang et al. fabricated epoxy/graphene nanocomposites with ~15 wt.% filler loading and obtained shielding effectiveness of 21 dB [11]. Wang et al. reported the 35 dB shielding effectiveness with the incorporation of hierarchical MWCNT-Fe_3_O_4_/Ag nanofiller (15 wt.%) [12]. It is obvious from these studies that CPCs with higher loadings of nanofillers from 15 to 61.5 wt.% have obtained greater shielding properties. However, achieving homogeneous nanofiller dispersion in a polymer matrix with hydrodynamics and viscoelastic properties to contend with is difficult. In addition, the mechanical properties of a polymer matrix may deteriorate with high filler loading. Therefore, it is challenging to obtain effective EMI shielding properties in polymer nanocomposites by loading low content of nanofillers.

Recently, the combination of multiphase polymer blend (polyblend) has been adopted to develop electrically conducting nanocomposites with low filler content, overcoming the problems of poor processability and deteriorating mechanical properties. In these polyblends, different phase structures with different filler localization can be possible, which is used to construct electrically conductive networks with low nanofiller loading. To form conductive networks with low percolation, it is necessary that the nanofiller be localized at the interface of the separated polymer phases in co-continuous or phase inversion polyblend with the balanced mechanical properties of two integrated polymers [13]. When the polyblend system contains a thermosetting polymer such as diglycidyl ether of bisphenol A (DGEBA), the curing reaction can control the localization of the nanofiller at the interface of two immiscible polymers [14]. Other than the thermodynamics of curing reaction, various kinetic and rheological factors also influence the final phase structure of the polyblend and preferential localization of fillers.

In the past few years, there have been various reports on selective localization of nanofiller in the co-continuous polyblend of epoxy and other amphiphilic block co-polymers [15,16] that are simply controlled by curing reaction-induced phase separation (CRIPS) [17,18,19,20]. However, there are fewer reports regarding using phase-separated structures to synthesize materials with low loadings of selectively localized conductive fillers to achieve favorable EMI shielding properties [21,22]. The preparation of rigid nanofiller foam that can withstand the hydrodynamic forces of resin infusion is not an easy task, and further practical applicability for complex geometries is difficult. Hence, a simple processing technology to develop thermosetting resin-based effective EMI shielding materials with low loading of conducting filler is extremely important.

In this work, a DGEBA/polyetherimide (PEI) polyblend system was prepared. The CRIPS technique facilitated the selective localization of reduced graphene oxide (RGO) at the interface of two immiscible phases. The resultant double-percolated conductive networks of RGO in co-continuous or inversion phase structure of DGEBA/PEI polyblends helped to improve the electrical conductivity of nanocomposites at a low concentration of RGO. Furthermore, the effects of blending and selective localization of RGO on the thermomechanical and EMI shielding properties of nanocomposites were investigated. This study provides an interesting technique for developing low nanofiller loading polyblend nanocomposites with balanced electrical, thermomechanical, and EMI shielding properties. These multifunctional properties contribute novel three-dimensional DGEBA/PEI/RGO nanocomposites for effective aerospace structural materials and microelectronics applications.

## 2. Experimental Section

### 2.1. Materials

The DGEBA oligomer was provided by Dow Chemical Co., Midland, Michigan, MI, USA, DER 332 with an epoxide equivalent weight of 172–176 g/eq. PEI (Ultem^®^1000, *T_g_* ≈ 217 °C) was purchased from Goodfellow Co., Huntingdon, UK. Commercial graphene oxide (GO) was provided by Standard Graphene Co., Ulsan, Korea. N,N-dimethylbenzylamine (DMBA) and methyl tetrahydrophthalic anhydride (Me-THPA) were supplied from Shanghai Reagent Co., Shanghai, China. N,N-Dimethylformamide (DMF), ethanol (C_2_H_5_OH), formamide (FA), glycerol (GL), hydrazine monohydrate (98%), and methylene dichloride (CH_2_Cl_2_) were purchased from Sigma-Aldrich, St. Louis, MO, USA. All reagents were used without further purification.

### 2.2. Synthesis of RGO

The preparation of RGO was according to a previous publication [14]. The RGO suspension was then filtered and washed by DMF for three times to remove the unreacted reducing agent (i.e., hydrazine). Finally, the RGO flakes were dried in the vacuum oven at 150 °C for 24 h to evaporate the residual solvent completely. These free-standing RGO papers were thus obtained for morphological and X-ray photoelectron spectroscopy (XPS) analysis.

### 2.3. Fabrication of DGEBA/RGO/PEI Nanocomposites

**Figure 1:**
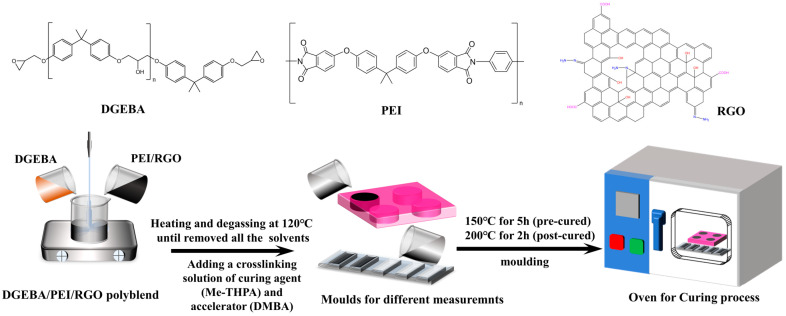
Schematic of the fabrication of DGEBA/PEI/RGO nanocomposites.

The nanocomposites with RGO were prepared using the solvent casting technique. Initially, PEI was dissolved in CH_2_Cl_2_ with magnetic stirring. A homogeneous suspension of RGO (0.4 mg/mL) was then added into PEI solution and stirred at room temperature. Subsequently, DGEBA oligomer was added into the PEI/RGO mixture. The mixture was stirred at 90 °C for 2 h to remove most of the solvent and degassed in the vacuum oven at 120 °C for 12 h to completely remove the residual solvent. Finally, a crosslinking solution of DMBA and Me-THPA was added to the degassed polyblend (DGEBA: crosslinking solution = 1:0.8) and stirred at 110 °C. After pouring into the molds, the polyblend was then pre-cured at 150 °C for 5 h and post-cured at 200 °C for 2 h. The compositions of DGEBA, RGO, and PEI are given in Table S1. The DGEBA/PEI/RGO nanocomposites were designated as DP*n*_1_R*n*_2_ where D, P, *n*_1_, R, and *n*_2_ represent DGEBA, PEI, the weight percentage of PEI in DGEBA, the RGO, and the weight percentage of RGO in the DGEBA/PEI/RGO nanocomposites, respectively. For comparison, samples including neat DGEBA, nanocomposites DR1, DR1.5, DR2, DR2.5, DR3, DP25, and DP30 were prepared using a similar casting technique. A schematic of the fabrication process is shown in Figure 1.

### 2.4. Measurement and Characterization

Transmission electron microscope (TEM, H-8100, Hitachi High-Tech Co., San jose, CA, USA) and scanning electron microscope (SEM, S-3400N, Hitachi High-Tech Co., San jose, CA, USA) analyses were used to observe the morphology of the fillers. FETEM measurements were performed by using a FETEM (Tecnai G^2^ F20 X-Twin, FEI Co., Hillsboro, OR, USA) operated at an accelerating voltage of 200 kV. Ultrathin films (thickness: 100–200 nm) of the nanocomposite samples were prepared using an ultra-microtome (RMC CR-X, Boeckeler Instruments Co., Tucson, AZ, USA) equipped with a glass knife for FETEM observation. Raman spectra were performed by a Raman spectrometer (DXR™ 3, Thermo Fisher Scientific Inc., Woodward, Austin, AZ, USA). XPS analysis was performed on an ESCALAB 250XI (Thermo Fisher Scientific Inc., Woodward, Austin, AZ, USA) system using a monochromatic Al-Kα X-ray source (Kα 1486.6 keV) and a CLAM-2 hemispherical analyzer for electron detection. Fourier transform infrared (FTIR) spectra were recorded using an FT-IR (Nicolet™ iS™ 5, Thermo Fisher Scientific Inc., Woodward, Austin, AZ, USA) and attenuated total reflectance (ATR) accessory in the range of 4000–400 cm^−1^ at room temperature. The specimens were prepared in disk-shaped silicone molds with 40 mm in diameter and 2 mm in thickness of the specimens and measured by using the four-point probe equipment (CMT-100MP, AIT Co., Gyeonggi, Korea) for electrical properties measurement. The thermomechanical properties of the specimens were attained by DMA (Q800, TA Instruments Co., Lukens Dr, New Castle, DE, USA) in the single cantilever mode at a frequency of 1 Hz. The temperature ranges applied were from 30 to 180 °C for neat DGEBA and from 30 to 230 °C for other nanocomposites at a heating rate of 3 °C/min. The samples were cast in 45 × 10 × 3 mm^3^ rectangular aluminum molds for DMA analysis. The contact angle (CA) measurements were conducted by a Phoenix 300 (SEO Co., Gyeonggi-do, Korea) using the sessile drop method at room temperature with 15–17 μL of deionized water (DI), glycerol (GL), and formamide (FA), respectively. Thermogravimetric analysis (TGA, Q50, TA Instruments Co., Lukens Dr, New Castle, DE, USA) was carried out under a nitrogen atmosphere at a heating rate of 10 °C/min. Differential scanning calorimeter (DSC, Q20 V24.10, TA Instruments Co., Lukens Dr, New Castle, DE, USA) measurements were performed under a constant nitrogen flow of 50 mL/min and at a heating rate of 20 °C/min. The EMI shielding effectiveness (SE) parameters of nanocomposites were measured by a vector network analyzer (E5071C Agilent Inc., Bensenville, IL, USA) in the frequency range of 8.2−12.4 GHz (X-band) at room temperature. The samples in 2 mm thickness were cut into rectangle plates of 22.9 × 10.2 mm^2^ to fit the waveguide sample holder.

## 3. Results and Discussion

### 3.1. Morphological Properties of RGO

**Figure 2:**
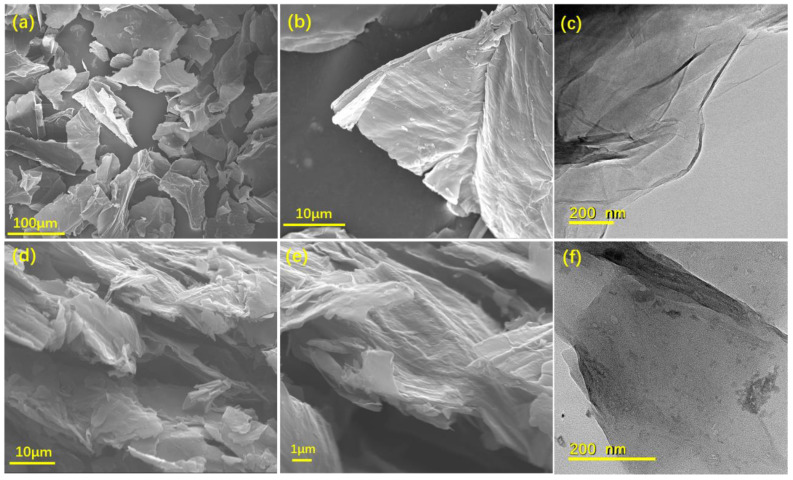
SEM micrographs of pristine GO at (**a**) low and (**b**) high magnification. (**c**) TEM micrograph of GO. SEM micrographs of RGO flakes at (**d**) low and (**e**) high magnification. (**f**) TEM micrograph of RGO.

SEM micrographs of GO and RGO flakes are shown in Figure 2a,b and Figure 2d,e, respectively. Figure 2a,b shows that the pristine GO is randomly aggregated in wide size distribution. Figure 2d,e illustrates that RGO has many wrinkles and crumples after hydrazine reduction, and they are entangled with each other. TEM micrographs of GO and RGO are shown in Figure 2c,f. TEM micrographs of GO and RGO represent the wrinkled structure with a few layers stacking, and Raman, FT-IR, and XPS analysis were also conducted to estimate the actual structural changes between GO and RGO before RGO was introduced into polyblends to prepare nanocomposites.

### 3.2. Physical and Chemical Properties of RGO

**Figure 3:**
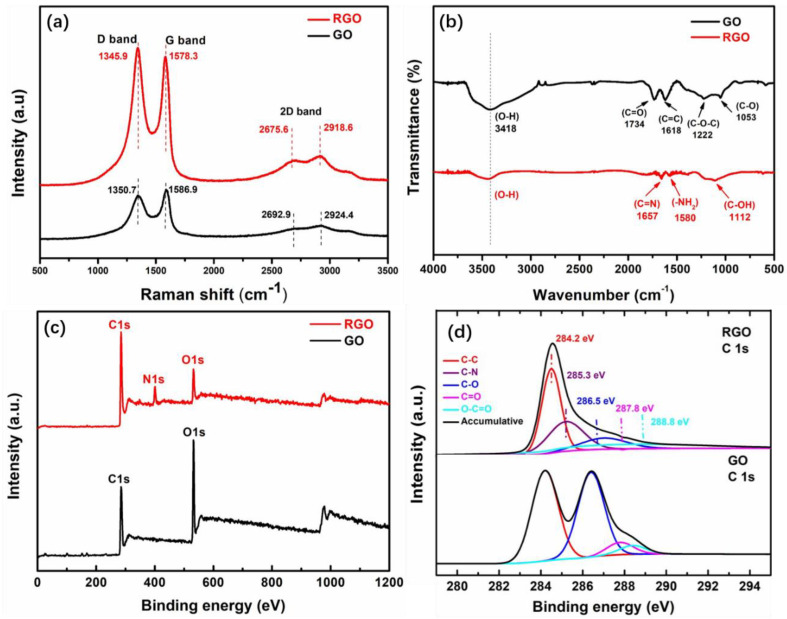
(**a**) Raman and (**b**) FT-IR spectra of RGO flakes and GO. (**c**) Wide-scan, and (**d**) C 1s high-resolution core-level of XPS spectra for GO and RGO flakes.

Raman analysis provides information regarding the inelastic scattering by molecules irradiated by the monochromatic excitation source, thereby elucidating the structural properties of a material. Figure 3a represents the Raman spectra of pristine GO and RGO. Two fundamental vibrations can be observed between 1200 and 1600 cm^−1^ from both GO and RGO. The first D vibration band that is associated with κ-point photon breathing mode of A_1g_ symmetry of GO and RGO appears at 1350.7 and 1345.9 cm^−1^, respectively. The G vibration band that is associated with E_2g_ phonons of sp^2^ hybridized carbon of GO and RGO is found at 1586.9 and 1578.3 cm^−1^, respectively [23,24]. The intensity ratio of the D band and G band (I_D_/I_G_) increases from 0.909 to 1.045 after hydrazine reduction of GO, due to the sp^2^ carbon cluster. The higher intensity of RGO suggests the reduction of oxygenated groups and the presence of more isolated graphene domains compared to GO [25]. Due to the multilayer structure, the Lorentzian peaks of the 2D band for GO and RGO appear at 2692.9 and 2675.6 cm^−1^, respectively. Moreover, the increased intensity of the 2D band with a slight shift to lower frequencies in RGO suggests inhibited stacking due to the reduction of oxygenated functional groups.

Figure 3b shows the FT-IR spectra of GO and RGO. The presence of intense peaks at 1734 cm^−1^ (from C=O stretching), 1222 cm^−1^ (from C–O–C stretching), 1053 cm^−1^ (from C–O stretching) and a broad band at ~3418 cm^−1^ (from hydroxyl groups) implies the presence of oxygenated functional groups in GO [26,27]. The disappearance of oxygeneous moieties along with the resultant nitrogenous groups from hydrazine treatment clearly indicates the successful reduction of pristine GO.

XPS analysis can provide information based on X-ray induced photoemission. In the case of RGO, a new peak appears at ~400 eV, which corresponds to the N1s component [28] that results from the hydrazine reduction of GO (Figure 3c). Furthermore, after reduction, the intensity ratio of peaks C1s/O1s for GO increases abruptly from 1.2 to 10.4, which is related to the quantitative information of the reduction of GO. In Figure 3d, GO contains a wide range of oxygenated functional groups, such as epoxide, carbonyl, and carboxyl groups [29,30], which are assigned at 286.5, 287.8, and 288.8 eV, respectively. In comparison with GO, peaks corresponding to oxygen-containing functional groups are significantly decreased in RGO after hydrazine reduction indicates that most of the oxygenated functional groups were removed [29,31]. Additionally, a new peak appears at 285.3 eV in RGO, which is the C–N bond ascribed to the hydrazine reduction [32,33]. A high-resolution core-level of the N1s spectrum shows the –C–NH_2_ bond at 399.9 eV, as shown in Figure S1. Additionally, the absence of the peak at ~398 eV corresponding to the N–N bond shows that the residual hydrazine (NH_2_–NH_2_) is below the detection limit of XPS. Therefore, the XPS results are consistent with those from FT-IR analysis, indicating that the reduction of GO into RGO is successful.

### 3.3. Morphologies and Chemical Properties of DGEBA/PEI/RGO Nanocomposites

The morphologies of the DP*n*_1_ polyblends and the localization of RGO in DP*n*_1_R*n*_2_ nanocomposites were observed by FETEM. Figure S2 exhibits FETEM micrographs of DP30, in which white and dark domains represent the DGEBA-rich and PEI-rich phases, respectively. The PEI-rich phase forms an inversion continuous network, while DGEBA appears as a small iso-island in the PEI-rich network. The size of PEI-rich ligaments lies in the range of 50 to 400 nm, but size distribution indicates an average size of ~55 nm, which is narrow for developing the conducting network of RGO in a polyblend system.

**Figure 4:**
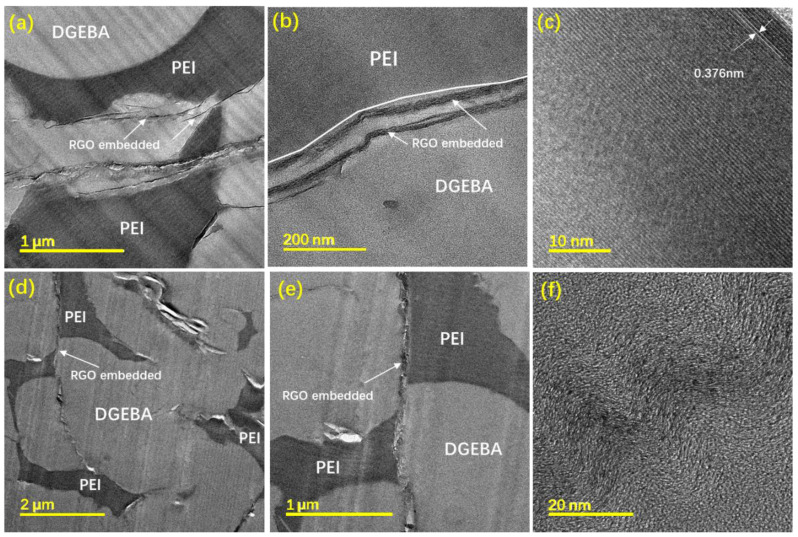
The FETEM micrographs of the DP25R3 nanocomposite at (**a**) lower and (**b**) higher magnification, (**c**) HRTEM micrograph of RGO in DP25R3, (**d**,**e**) DP30R3 nanocomposite with inversion morphology, and (**f**) HRTEM micrograph of RGO in DP30R3.

Figure 4a,d represents the inversion phase structure of DP25R3 and DP30R3, respectively. The phase structure of DP25R3 is changed from the co-continuous phase morphology of DP25 to the inversion phase morphology [14]. This change may be caused by the amino groups from RGO, which stimulate the CRIPS [19]. Figure 4b shows a double-percolated structure of DP25R3 formed by RGO selective localization at the interface and close to the DGEBA domain. The presence of RGO at the interface is confirmed by the HRTEM of DP25R3 with the fringe pattern at the interface. The d-spacing value of RGO flakes is 0.376 nm in Figure 4c. Figure 4e shows that most of the crumpled surfaces of RGO flakes distribute selectively at the interface between DGEBA-rich and PEI-rich domains for nanocomposite DP30R3. Figure 4f exhibits the wavy fringe pattern structure of RGO that is observed at the interface of the magnified micrograph [34,35].

**Figure 5:**
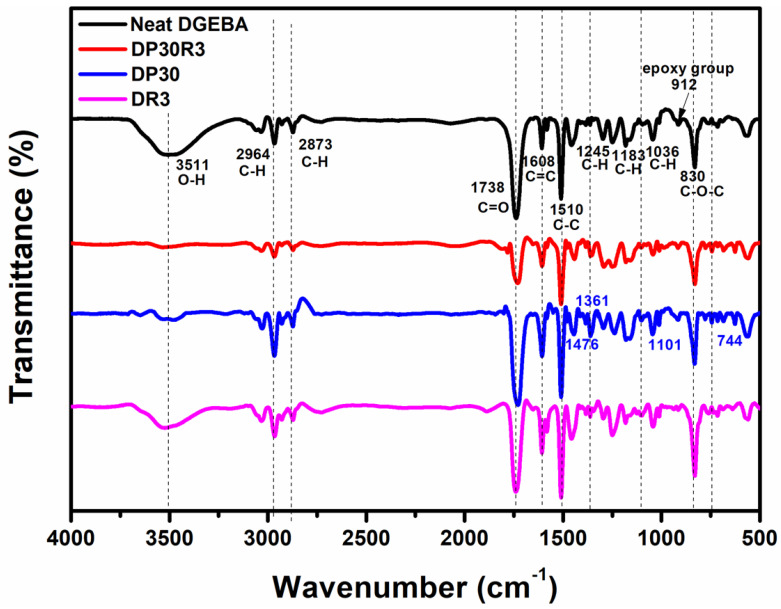
The FT−IR spectra of DR3, DP30, DP30R3, and neat DGEBA.

FT-IR analysis was conducted on neat DGEBA, polyblend DP30, nanocomposites DR3 and DP30R3, respectively, and this spectral information is presented in Figure 5. The peaks of neat DGEBA at ~2964, 2873, 1608, 1510, 912, and 830 cm^−1^ correspond to the C–H stretching of CH_2_, C–H stretching of aromatic and aliphatic, C=C stretching of aromatic rings, C–C stretching of aromatic, C–O stretching of oxirane group, and O–C–O stretching of oxirane group, respectively [36,37]. Meanwhile, the peaks at ~1245, 1183, and 1036 cm^−1^ belong to different aliphatic and aromatic C–H vibrations [38].

The peaks of DP30 at ~1476, 1361, 1101, and 744 cm^−1^ correspond to aromatic ring stretching, C–N stretching (in phthalimide rings), Ar–O–Ar stretching, and C–N bending (in phthalimide rings) vibrations, respectively [39,40], which are not visible in neat DGEBA or DR3. In comparison with DP30, however, DP30R3 does not show a significant decrement in the peak intensities at ~1658 and 1573 cm^−1^, which are associated with C=N and –NH_2_ stretching, respectively, and are formed by hydrazine reduction of RGO [14], due to the low content of RGO in the polyblend. The PEI chains might more efficiently interact with RGO through non-bonding interactions such as electrostatic, dipole, and π-π stacking interactions between PEI and RGO basal plane [41].

### 3.4. Prediction for Selective Localization of RGO

Several studies have used different complex parameters to solve the selective localization of a kind of nanofiller in an immiscible polyblend, even if the analysis of localization involves many factors, such as the thermodynamics, kinetics, fluid dynamics, viscosity ratio, and phase separation [42,43,44], and considering the wettability parameter is still an efficient approach to estimate it.

To assess the surface tension of components, CA measurements with the geometrical mean (GM) method [45] was conducted, and then the surface tension was calculated using the Owens–Wendt equation combined with the Young equation [46] as follows:

(1) $$\gamma_{L}\left(1+\cos\theta \right)=2\sqrt{\gamma_{S}^{d}\gamma_{L}^{d}}+2\sqrt{\gamma_{S}^{p}\gamma_{L}^{P}}$$

where γ_S_ and γ_L_ are the surface tensions of the solid and liquid compound, respectively, and d and p are the dispersive and polar portions of surface tension, respectively.

Figures of the contact angles are shown in the Supplementary Materials (Figure S3). The values of static contact angles and parameters are shown in Table S2. The surface tensions were calculated by GM approaches, as shown in Table S3. The interfacial tension (γ_A–B_) can be calculated by the harmonic mean (HM) Equation (2) [45] and the GM Equation (3) [46] as follows:

(2) $$\gamma_{A-B}=\gamma_{A}+\gamma_{B}-2\left(\sqrt{\gamma_{A}^{d}\gamma_{B}^{d}}+\sqrt{\gamma_{A}^{p}\gamma_{B}^{p}}\right)$$

(3) $$\gamma_{A-B}=\gamma_{A}+\gamma_{B}-4\left(\gamma_{A}^{d}\gamma_{B}^{d}/(\gamma_{A}^{d}+\gamma_{B}^{d})+\gamma_{A}^{P}\gamma_{B}^{p}/(\gamma_{A}^{P}+\gamma_{B}^{P})\right)$$

The wetting coefficient (ω_a_) first proposed by Sumita et al. [47] is generally applied for predicting fillers localization [48,49,50]. The ω_a_ can be calculated by Young’s equation:

(4) $$\omega_{a}=\gamma_{R-P}-\gamma_{R-D}/(\gamma_{D-P})$$

where γ_R-P_, γ_R-D_, and γ_D-P_ are the interfacial tensions between the RGO and PEI phase, between the RGO and DGEBA phase, and between the DGEBA and PEI phase, respectively. The prediction is based on the value of ω_a_. RGO will localize in the PEI phase preferentially if ω_a_ < −1, or at the interface between DGEBA and PEI if −1 < ω_a_ < 1, or even in the DGEBA phase if ω_a_ > 1.

The interfacial tension and wetting coefficients for nanocomposites DGEBA/PEI/RGO were calculated using the HM and GM methods (Table 1). The wetting coefficients were found to be −1 < ω_a_ = 0.998 < 1 using the HM method and ω_a_ = 1.060 > 1 using the GM method, which indicates that the RGO may localize selectively at the interface and have an affinity toward the DGEBA phase in DGEBA/PEI/RGO systems. The values of ω_a_ from both the harmonic and geometric methods are close to 1, indicating the two possibilities of selective localization for RGO according to different RGO content. In our previous work with 0.5 wt.% RGO in nanocomposites [14], the prediction using the harmonic method showed that RGO selectively localized at the interface. However, by increasing the content of RGO to 3 wt.% in this work, a few RGO entered the DGEBA phase close to the interface, as predicted by the geometric method, due to the limitation of the interface and the strong interfacial tension of DGEBA. This prediction is consistent with the FETEM micrographs presented in Figure 4.

### 3.5. Electrical Properties of Nanocomposites

**Figure 6:**
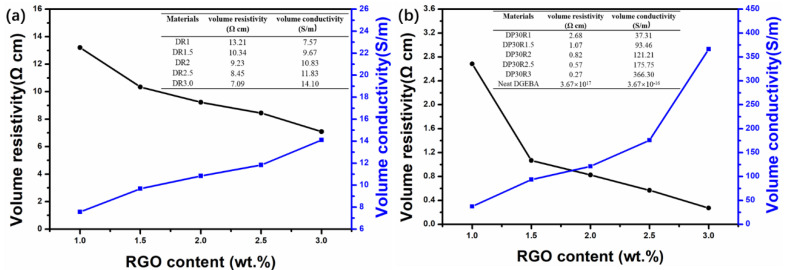
The volume resistivity and volume conductivity of (**a**) DR*n*_2_ and (**b**) DP30R*n*_2_ nanocomposites with varying RGO content.

Figure 6a,b shows the electrical properties of nanocomposites DR*n*_2_ and DP*n*_1_R*n*_2_, respectively. Compared to DR*n*_2_, DP*n*_1_R*n*_2_ has higher electrical conductivity per RGO content, as shown in Figure 6b. The electrical conductivity of nanocomposite DP30R3 reaches 366.3 S/m, which is almost 26 times that of nanocomposite DR3 (14.1 S/m). It has been reported that the selective localization of nanofiller would play a crucial role in the electrical properties of nanocomposites [51,52]. When a conductive nanofiller is selectively localized at the interface of two immiscible polymers system, it facilitates the formation of a conductive pathway in the polyblend at minimal nanofiller content. From the electrical conductivity difference between DP30R3 and DR3, it is evident that the PEI in nanocomposite DP30R3 facilitates the selective localization of RGO at the interface and the formation of conductive networks. FETEM observations (Figure 4) show the morphology of DP30R3 polyblends with an inversion phase structure that is different from the morphologies of nanocomposites without PEI. The simultaneous curing reaction of DGEBA and phase separation between DP*n*_1_ according to the spinodal decomposition mechanism allows for the formation of a double-percolation RGO network structure. It is this double-percolation RGO network localizing at the interface between DGEBA and PEI that may cause significantly improved electrical conductivity.

From the electrical conductivity measurement of nanocomposites with different RGO contents, it has been seen that a high content of RGO is required to form the conductive networks. Moreover, in comparison with DR3, DP30R1 possesses a higher volume conductivity despite its lower RGO content (1 wt.%). This difference is likely caused by the incorporation of PEI in DGEBA, leading to a double percolation conductive RGO network formed at their interface. In other words, only a small amount of RGO is needed to form conductive pathways at the continuous interface and enough to achieve the insulator/conductor transition. Further, among the DP*n*_1_R*n*_2_ nanocomposites, DP30R3 exhibits the highest electrical conductivity (366.3 S/m), which is higher than those of other reported polyblends networks, even though it has a lower RGO loading (Table S4).

### 3.6. Thermal and Thermomechanical Properties of Nanocomposites

**Figure 7:**
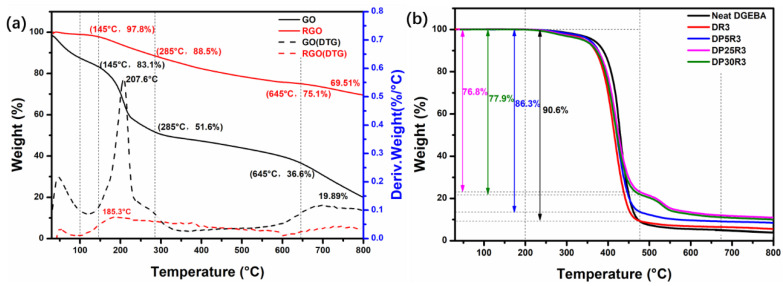
The TGA spectra of (**a**) GO and RGO, and (**b**) neat DGEBA, DR3, DP5R3, DP25R3, and DP30R3 under N_2_ atmosphere.

The chemical changes and the thermal degradation/stability of GO and RGO were investigated by TGA. Figure 7a represents the TGA and derivative thermogravimetric (DTG) curves of GO and RGO. RGO exhibits higher thermal stability than pristine GO over the entire temperature range of the measurement. The evaporation of water adsorbed at the hydrophilic surface of the GO sheets causes the DTG of GO with mass loss below 100 °C. [53]. GO shows two-step degradation, and the initial mass loss of GO at around 207 °C is ascribed to the decomposition of the labile oxygenous groups, such as epoxy, hydroxyl, and carbonyl [54]. The second step of the degradation (285–645 °C) is associated with the pyrolysis of residual oxygenous groups. RGO exhibits only ~12% weight loss up to 285 °C, which is much lower than GO in a similar temperature range. Finally, GO at 800 °C is only 19.9 wt.% left. After chemical reduction, the residue of RGO at 800 °C is significantly increased to 69.5 wt.%, which is much higher than that of pristine GO.

Figure 7b represents the TGA spectra of neat DGEBA along with nanocomposites. DP25R3 and DP30R3 exhibit two-stage degradation, whereas neat DGEBA, nanocomposite DR3 and DP5R3 with low PEI content (5 wt.%) all exhibit only one-stage degradation. For nanocomposites DP25R3 and DP30R3, the first stage of weight loss of ~77% happens from 200 to 457 °C and is ascribed to the decomposition of DGEBA and the non-aromatic part of PEI [55]. The second stage of weight loss around 12% occurs from 475 to 675 °C due to the decomposition of the aromatic part of PEI. The TGA data of samples are also summarized in Table S5.

The thermal and thermomechanical properties of the nanocomposites were analyzed using the DSC and DMA. The effects of selective localization of nanofillers in the nanocomposites on physical properties were studied.

**Figure 8:**
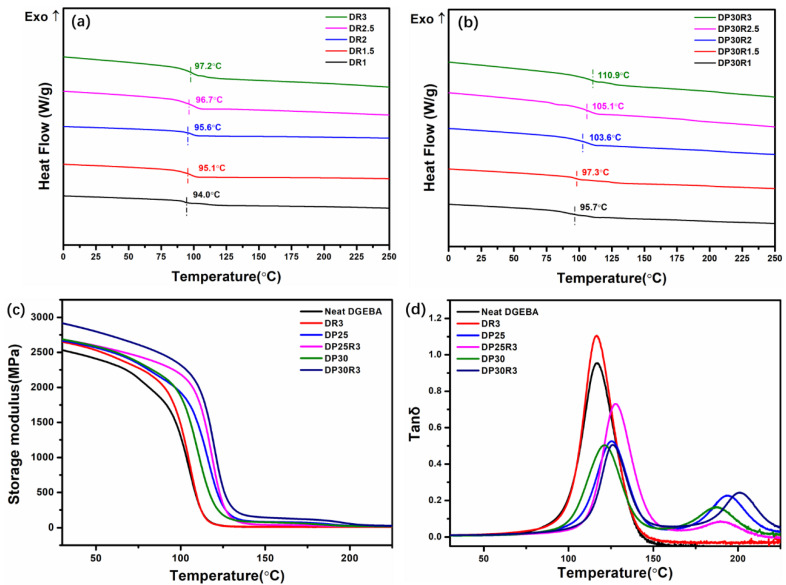
The DSC curves of (**a**) DR*n*_2_ and (**b**) DP30R*n*_2_. The DMA measurements of (**c**) storage modulus and (**d**) tan *δ* of the nanocomposites as a function of temperature.

The DSC analyses provide heat flow as a function of temperature. Figure 8a,b shows the effect of RGO content on the thermal properties of nanocomposites DR*n*_2_ and DP30R*n*_2_. It indicates that the *T*_g_ increases with increasing RGO content. The value of *T*_g_ increases from 93.3 °C for neat DGEBA to 97.2 °C for DR3, and to 110.9 °C for DP30R3. The increased *T*_g_ of DR3 is due to inhibition of the motion of DGEBA segments. For DP30R3, the change in internal energy of the nanocomposite due to the thermoplastic polymer PEI and nanofiller RGO is primarily responsible for the improved *T_g_* [19]. In addition, the value of *T_g_* for PEI is nearly 217 °C, which is much higher than that of DGEBA. Continuous PEI ligaments in the inversion phase structure will restrict the movement of DGEBA chains at elevated temperatures, which improves the *T_g_*. The values of *T_g_* are summarized in Table S6. Further, some PEI dissolves in DGEBA, leading to the higher *T_g_*. These improvements in *T_g_* are further confirmed by DMA.

DMA provides phase angle and deformation data (for calculation of storage modulus and tan δ) by applying stress or strain to specimens and analyzing the response. DMA can also reveal information about the viscoelastic behavior and thermomechanical properties of the nanocomposites. Compared to DP*n*_1_ polyblends and DR*n*_2_ nanocomposites, DP *n*_1_R *n*_2_ nanocomposites have a higher storage modulus at the same PEI content, as shown in Figure 8c, indicating that the synergistic effect of PEI and RGO provides a higher reinforcement. The highest value of *E*’ for DP30R3 reaches 2917 MPa in the glassy region and 1366 MPa in the rubbery region, which is an overall improvement of ~15% and ~1101% in comparison with that of neat DGEBA, and an improvement of ~10% and ~1041% over the corresponding values for DR3. The rubbery modulus increases with the addition of RGO nanofiller, indicating that the crosslinking densities of DP*n*_1_R*n*_2_ nanocomposites increase with RGO nanofiller content. The values of storage modulus and tan *δ* are summarized in Table S7.

A plot of tan *δ* versus temperature for DP*n*_1_, DR*n*_2_, and DP*n*_1_R*n*_2_ nanocomposites is shown in Figure 8d. Unlike the results of DSC, both DP*n*_1_ and DP*n*_1_R*n*_2_ show two transition peaks associated with the *T_g_* of the DGEBA-rich phase (*T_g_*_1_) and the PEI-rich phase (*T_g_*_2_). The increasing trend of *T_g_*_1_ is consistent with that from DSC measurements. In comparison with the *T_g_* of neat DGEBA (116.1 °C), the increase in *T_g_*_1_ for DR3, DP30, and DP30R3 is 1, 5, and 10.1 °C, respectively.

### 3.7. EMI Shielding Measurements

A material that attenuates the intensity of an electromagnetic (EM) wave and inhibits its transmission is referred to as an EMI shielding material, and its abilities are quantified as SE. Generally, there are three basic waves involved in the shielding of EM waves: reflection, absorption, and multiple reflections [56]. The SE_T_ of EMI shielding material can be represented as follows [57]:

(5) $${\mathrm{SE}}_{T}={\mathrm{SE}}_{A}+{\mathrm{SE}}_{R}+{\mathrm{SE}}_{M}$$

where SE_A_, SE_R_, SE_M_, and SE_T_ are the absorption loss, reflection loss, multi-reflection loss, and total shielding effectiveness, respectively. The SE_M_ of EMI shielding material can be expressed as follows:

(6) $${\mathrm{SE}}_{M}=10\log\left[1-2\times 10^{0.1{\mathrm{SE}}_{A}}\cos\left(0.235{\mathrm{SE}}_{A}\right)+10^{-0.25{\mathrm{SE}}_{A}}\right]$$

Equation (6) illustrates that SE_M_ is closely related to SE_A_, and it can be neglected when SE_A_ > 15 dB [58,59]. Thus, two main types of loss, SE_A_ and SE_R_, are considered in attenuating the incident electromagnetic radiation for EMI shielding material. SE_T_, SE_A_, and SE_R_ can be obtained by the scattering parameters (S_21_ and S_11_) measured by a vector network analyzer through the Equations (7)–(9) [60]:

(7) $${\mathrm{SE}}_{T}=-10\log{\left|S_{21}\right|}^{2}$$

(8) $${\mathrm{SE}}_{R}=-10\log\left(1-{\left|S_{11}\right|}^{2}\right)$$

(9) $${\mathrm{SE}}_{R}=-10\log\left(1-{\left|S_{11}\right|}^{2}\right)$$

Here, S_11_ and S_21_ denote the response at port 1 in response to a signal at port 1 and the response at port 2 in response to a signal at port 1, respectively.

**Figure 9:**
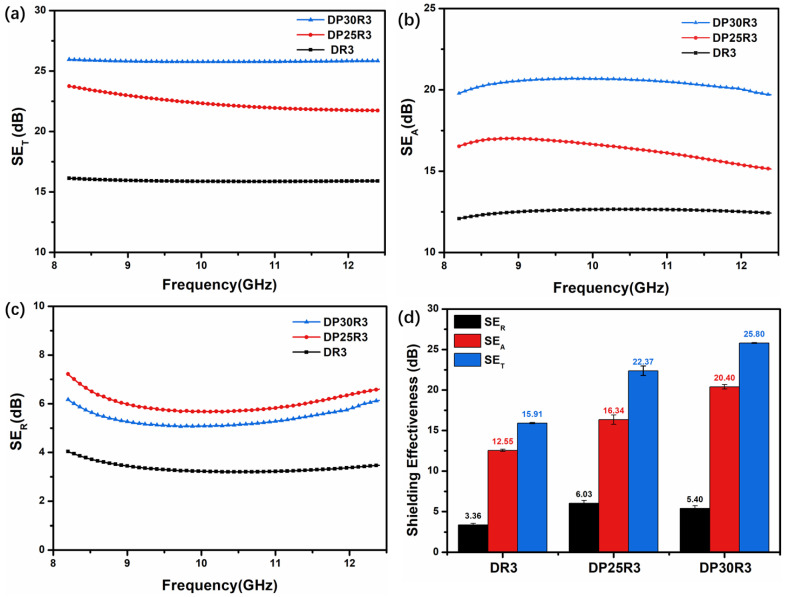
The EMI shielding effectiveness of DR3, DP25R3, and DP30R3 in the 8.2–12.4 GHz frequency range, (**a**) SE_T_, (**b**) SE_A_, (**c**) SE_R_, and (**d**) the average SE_T_, SE_A_, and SE_R_ in the X band.

In polymer nanocomposites, EMI shielding properties can be achieved by various means, such as introducing the conducting nanofillers, using a conducting polymer matrix, or by a combination of both. For polyblend shielding materials, phase structure is important. In particular, the continuous phase can play a crucial role in developing electrically conductive networks for the attenuation of electromagnetic waves. Figure 9a–d shows the EMI shielding effectiveness of DR3, DP25R3, and DP30R3. The SE_T_ of DR3 reaches ~15.9 dB, which is lower than those commercially adopted shielding materials (i.e., 20 dB). This indicates the inability of the low nanofiller content of 3 wt.% to develop uniform networks in the DGEBA matrix. When PEI is blended with DGEBA, a significant increase in shielding properties is observed. The average SE_T_ of DP25R3 in the inversion phase structure reaches ~22.4 dB with the help of an RGO nanofiller in the frequency range of 8.2–12.4 GHz. In this inversion phase structure, DGEBA-rich islands disperse in the thin continuous PEI-rich phase. Further, the selective localization of RGO at the interface improves the conducting losses and hence the overall shielding effectiveness. When the PEI reaches to 30 wt.%, the average SE_T_ of DP30R3 reaches the highest value of 25.8 dB in the same frequency range. The improved shielding effectiveness is due to the continuous phase of the PEI-rich phase, which provides more intensive nanofiller networks at the interface. The formation of the selectively localized RGO network can be further confirmed by comparing the absorption and reflection properties of DP25R3 and DP30R3. For nanocomposite DP30R3 (Figure 9b–d), the shielding effectiveness due to absorption (SE_A_) is about 20.4 dB, which is ~25% higher than that of nanocomposite DP25R3. In contrast, the shielding effectiveness due to reflection (SE_R_) for nanocomposite DP30R3 reaches 5.4 dB, which is 10% lower than that of nanocomposite DP25R3. This result may be caused by the decreased skin depth [61] due to the increase in the electrically conducting networks in DP*n*_1_R*n*_2_ nanocomposites and the high conductive losses induced by RGO.

To highlight the advantage of the CRIPS-based selective localization of conductive nanofiller for the preparation of low load nanocomposites for EMI shielding, a summary of previously reported EMI shielding composites is presented in Table S8. In comparison to the materials in other studies, DP*n*_1_R*n*_2_ (i.e., DP30R3) ternary nanocomposites present better shielding properties with lower filler loading. Additionally, the development of a double-percolated conductive network improves the absorption properties, which ultimately facilitates in improving the shielding effectiveness.

## 4. Mechanism

**Figure 10:**
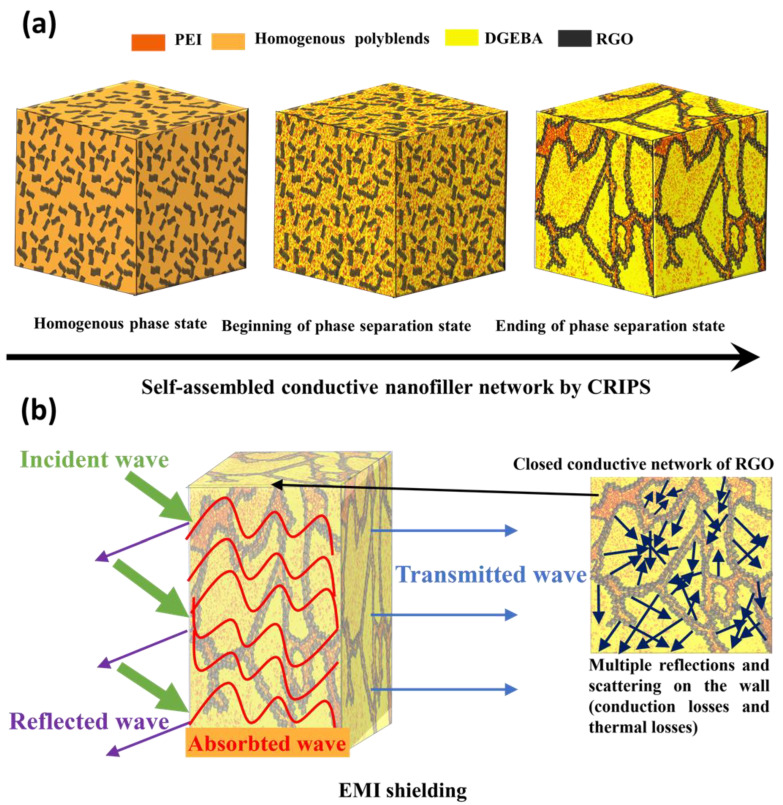
Schematic of (**a**) morphological evolution of double percolation, conductive DP*n*_1_R*n*_2_ nanocomposites and (**b**) the EMI shielding mechanism of DP*n*_1_R*n*_2_ nanocomposites.

Conventional polymers such as DGEBA and PEI are non-conductive and transparent to radiation. However, conductive nanofillers with good electromagnetic reflection and absorption properties will effectively enhance the shielding properties of composite materials when they are incorporated into a polymer matrix. For DP*n*_1_R*n*_2_ nanocomposites, not only does the combination of intrinsic electrical conductivity and absorption properties of RGO enhance EMI shielding, but also the self-assembly RGO networks via selective localization at the interface of polyblend DP*n*_1_ by CRIPS mechanism contributes to EMI shielding properties (Figure 9). Figure 10a shows the CRIPS mechanism of the DP*n*_1_R*n*_2_ nanocomposites. DGEBA oligomers begin with linear growth of the chains and then proceed with branching reactions by increasing the temperature. When their molecular weight reaches a critical value, phase separation occurs. At the beginning of phase separation, the DGEBA did not form the crosslinking network. As the reaction continued, and the temperature crossed the *T_g_* of DGEBA and crosslinking density reached to a critical value, and the cross-linked DGEBA were formed in the DGEBA/PEI polyblend [62]. Meanwhile, the crosslinking degree of DGEBA increased [63]. For 25 and 30 wt.% PEI in the presence of 3 wt.% RGO, an inversion phase structure formed according to spinodal decomposition behavior. PEI surrounds DGEBA oligomers with RGO selectively localized at the interface. The DP*n*_1_ polyblend system also helps RGO to construct a double-percolation structure at a much lower RGO content than has been demonstrated in other ternary polyblend nanocomposites.

Figure 10b shows the incident electromagnetic waves entering the DP*n*_1_R*n*_2_ nanocomposites divided into four wave pathways: the reflected wave, absorbed wave, transmitted wave, and waves repeatedly reflected and scattered between inner interfaces. EMI shielding includes mainly the reflection and absorption mechanisms [60]. The reflection mechanism is caused by mobile charge carriers such as electrons or holes bouncing off the shielding material, whereas the absorption mechanism is due to the absorption of radiation by electric and/or magnetic dipoles with a high dielectric constant. In these nanocomposites, the incident waves were absorbed due to destructive interference of EM waves, the closed-cell network of RGO, conducting losses, and thermal losses by RGO, as shown in Figure 10b. These double-percolated conductive networks of RGO in DP*n*_1_I polyblend form closed cell structures that produce superior absorption losses, which is consistent with the EMI shielding measurements of DP*n*_1_R*n*_2_ nanocomposites.

## 5. Conclusions

In summary, DGEBA/PEI/RGO ternary nanocomposites were successfully fabricated using a solution blending followed by the casting technique. The successful reduction of pristine GO into RGO was confirmed by Raman, FT-IR, XPS, SEM, and TEM. The decomposition of DGEBA/PEI/RGO followed a CRIPS mechanism. Polyblend DP30 and nanocomposite DP30R3 formed the inversion phase structure with separated DGEBA-rich phase surrounded by the continuous PEI-rich phase. The introduction of RGO facilitated the formation of the inversion phase structure in nanocomposite DP25R3 from the co-continuous phase structure of polyblend DP25. For both nanocomposites DP30R3 and DP25R3, RGO selectively localized at the interface between DGEBA and PEI, which was consistent with predictions from harmonic and geometric methods.

The maximum electrical conductivity of the optimized DP30R3 nanocomposite reached ~366 S/m, which is superior to that of DR3 (~14 S/m). This result clearly implies that only 3 wt.% of RGO is sufficient for developing a well-established conducting filler network in a polyblend nanocomposite, which would not be possible in a matrix of only DGEBA. This nanofiller network not only improved the inherent electrical properties of the polyblend but also improved the thermal and thermomechanical properties, which were confirmed by TGA, DSC, and DMA analysis. From the DSC and DMA measurements, the *T_g_* of the DGEBA-rich phase for nanocomposite DP30R3 was found to be almost 13.7 °C (DSC) or 9 °C (DMA) higher than that of polyblend DR3, clearly suggesting the restricted motion of chain segments at the interface of the two immiscible polymers. Finally, the applicability of these electrically conducting DGEBA/PEI/RGO nanocomposites for EMI shielding was analyzed under the X band frequency range. It was found that the effective EMI shielding of DGEBA/PEI/RGO reached 25.8 dB, of which ~80% was absorbed as conduction losses, thermal losses, and destructive interference losses. Therefore, these multifunctional RGO nanocomposites with low conductive filler loading provided a possibility in acting as effective aerospace structural materials.

## Figures and Tables

**Table 1:** The interfacial tension (γ_pair_) and ω_a_ were obtained by different methods.

Nanocomposites	Component Pair	γ_pair_(mN/m) Harmonic	γ_pair_(mN/m) Geometric	ω_a_	Predicted Localization of RGO
DGEBA/PEI/RGO	DGEBA/PEI	3.14	1.63	−1 < 0.998 < 1	Interface (HM)
	DGEBA/RGO	4.42	2.37	1.060 > 1	DGEBA phase (GM)
	PEI/RGO	1.28	0.65		

## Data Availability

The data presented in this study are available on request from the corresponding author (J.S.C.).

## Supplementary Materials

The following are available online at https://www.mdpi.com/article/10.3390/polym14050967/s1, Figure S1: XPS spectra of RGO flakes of N 1 s high-resolution core-level. Figure S2: FETEM images of (a) DP30 in low magnification and the histogram of the relative frequency versus the PEI thickness exhibiting the PEI thickness distribution in the figure (inset), (b) DP30 in high magnification in which the dark domains are PEI-rich phases and bright domains are DGEBA-rich phases, respectively. Figure S3: The contact angle images of samples: (a) DI on DGEBA, (b) DI on PEI, (c) DI on RGO, (d) GL on DGEBA, (e) GL on PEI, (f) GL on RGO, (g) FA on DGEBA, (h) FA on PEI, and (i) FA on RGO. Figure S4. The OM micrographs of (a) DP25R3 and (b) DP25. The dark yellow domains represent the PEI-rich phase, bright yellow domains represent the DGEBA-rich phases, and black sheets represent RGO. Table S1: Formulation of samples. Table S2: The surface tension parameters (in mJ/m^2^) of test liquids and static contact angles for DGEBA, PEI, and RGO. Table S3: The surface tension parameters (in mJ/m^2^) for the free surfaces of DGEBA, PEI, and RGO. Table S4: Comparison of electrical properties of previously published filler ternary polyblend systems with those of the systems in the current work. Table S5: The TGA spectra of samples under N_2_ atmosphere. Table S6: The glass transition temperature *T_g_* (taken as the intersection of the extrapolation of baseline with the extrapolation of the inflexion) of samples. Table S7: The storage modulus and *T_g_* of various samples. Table S8: Comparison of EMI shielding performance with other composites.
