# Supplementary material for: Enhanced Electromagnetic Interference Shielding Properties of Immiscible Polyblends with Selective Localization of Reduced Graphene Oxide Networks
Source: Polymers (Basel). 2022 Feb 28;14(5):967. doi: 10.3390/polym14050967 (PMC8912556; doi:10.3390/polym14050967)
Supplement: Supplementary file 1 [file polymers-14-00967-s001.zip › polymers-1584532-supplementary.pdf]

Supplementary Materials

Enhanced Electromagnetic Interference Shielding Properties of Immiscible Polyblends with Selective localization of Reduced Graphene Oxide Networks

Yiming Meng [a,b], Sushant Sharma [a], Jin Suk Chung [a,*], Wenjun Gan [b,*],Seung Hyun Hur [a] and Won Mook Choi [a]

[a]School of Chemical Engineering, University of Ulsan, Daehakro 93, Namgu, Ulsan 44610, Republic of Korea; 15026557980@163.com (Y.M.); sushant.nplindia@gmail.com (S.S.); shhur@mail.ulsan.ac.kr (S.H.H.); wmchoi98@ulsan.ac.kr (W.M.C.)

[b]Department of Macromolecular Materials and Engineering, College of Chemistry and Chemical Engineering, Shanghai University of Engineering Science, Longteng Road 333, 201620, Shanghai, P.R. China

****Correspondence: jschung@mail.ulsan.ac.kr (J.S.C); wjgan@sues.edu.cn (W.G.); Tel: +82-052-259-2249*

Table S1. Formulation of samples.

Samples	DGEBA (phr)[1]	PEI (phr)[1]	RGO (phr)[1]	C–A solution
DGEBA	100	0	0	80
DR1	100	0	x/ (100+0+x) =1.0%	80
DR1.5	100	0	x/ (100+0+x) =1.5%	80
DR2	100	0	x/ (100+0+x) =2.0%	80
DR2.5	100	0	x/ (100+0+x) =2.5%	80
DR3	100	0	x/ (100+0+x) =3.0%	80
DP25	100	25	0	80
DP30	100	30	0	80
DP5R3	100	5	x/ (100+5+x) =3.0%	80
DP25R1	100	25	x/ (100+25+x) =1.0%	80
DP25R1.5	100	25	x/ (100+25+x) =1.5%	80
DP25R2	100	25	x/ (100+25+x) =2.0%	80
DP25R2.5	100	25	x/ (100+25+x) =2.5%	80
DP25R3	100	25	x/ (100+25+x) =3.0%	80
DP30R1	100	30	x/ (100+30+x) =1.0%	80
DP30R1.5	100	30	x/ (100+30+x) =1.5%	80

DP30R2	100	30	x/ (100+30+x) =2.0%	80
DP30R2.5	100	30	x/ (100+30+x) =2.5%	80
DP30R3	100	30	x/ (100 + 30+x) =3.0%	80

[1]phr: parts per hundred in the DGEBA, x: RGO (phr), C-A solution: a solution of Me-THPA and DMBA.

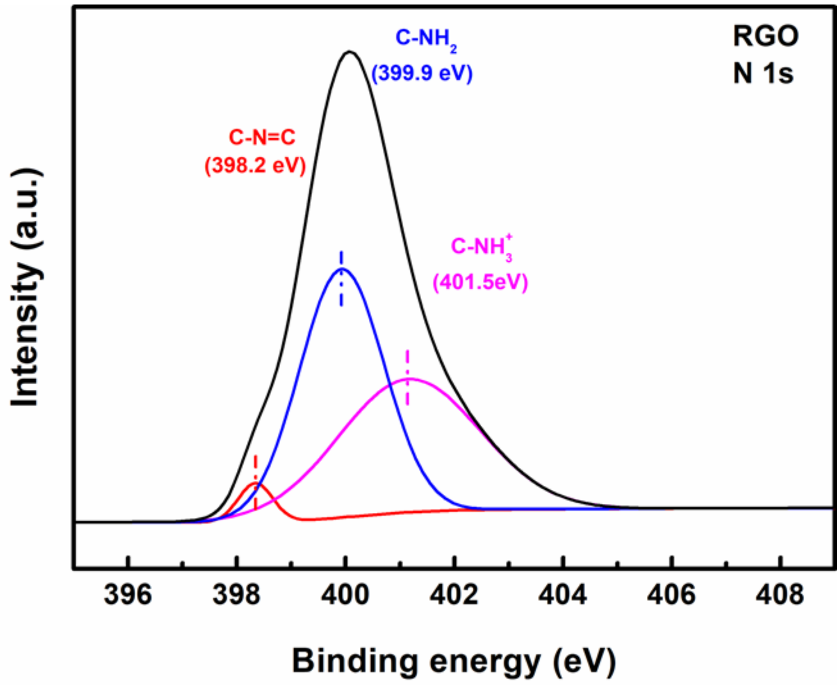

Figure S1. XPS spectra of RGO flakes of N 1 s high-resolution core-level.

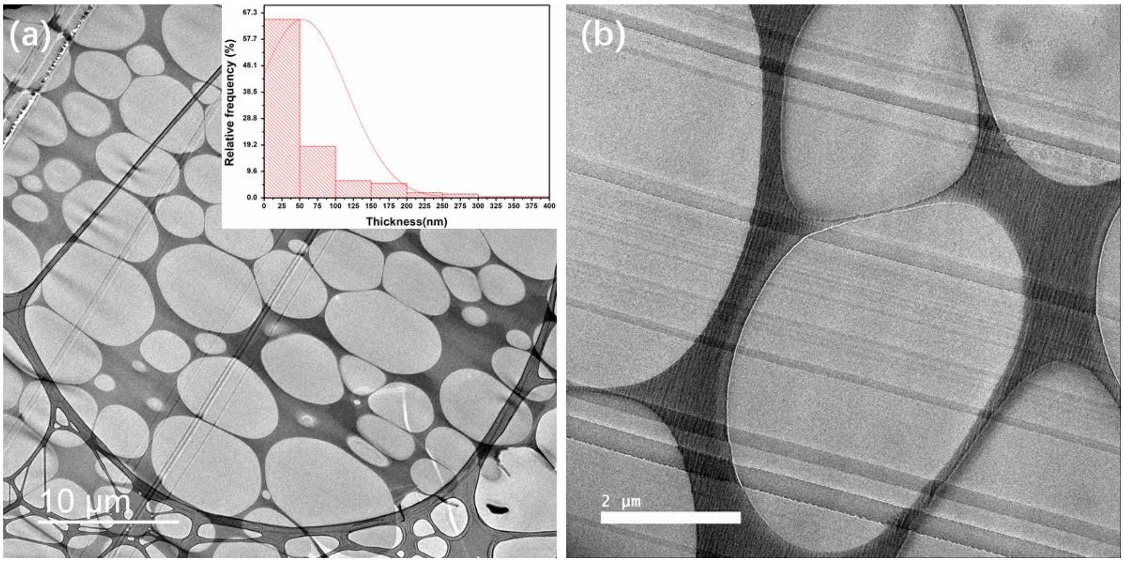

Figure S2. FETEM images of (a) DP30 in low magnification and the histogram of the relative frequency versus the PEI thickness exhibiting the PEI thickness distribution in

the figure (inset), (b) DP30 in high magnification in which the dark domains are PEI-rich phases and the bright domains are DGEBA-rich phases.

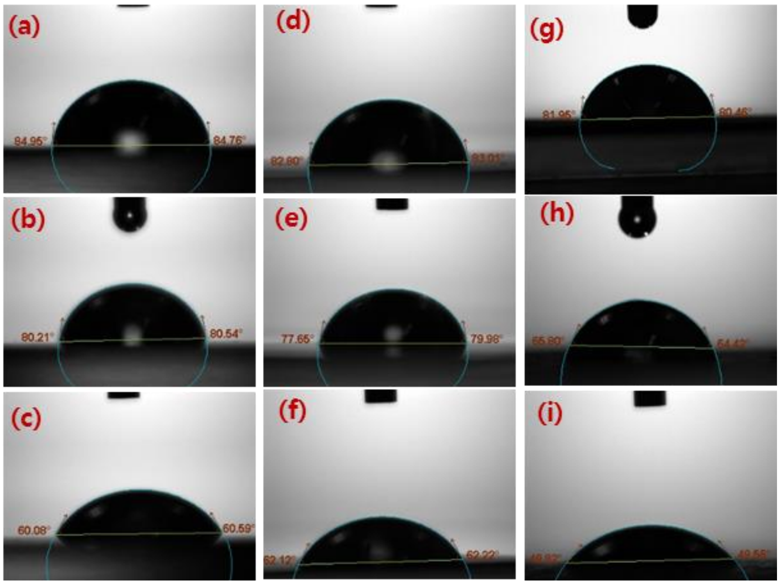

Figure S3. The contact angle images of samples: (a) DI on DGEBA, (b) DI on PEI, (c) DI on RGO, (d) GL on DGEBA, (e) GL on PEI, (f) GL on RGO, (g) FA on DGEBA, (h) FA on PEI, and (i) FA on RGO.

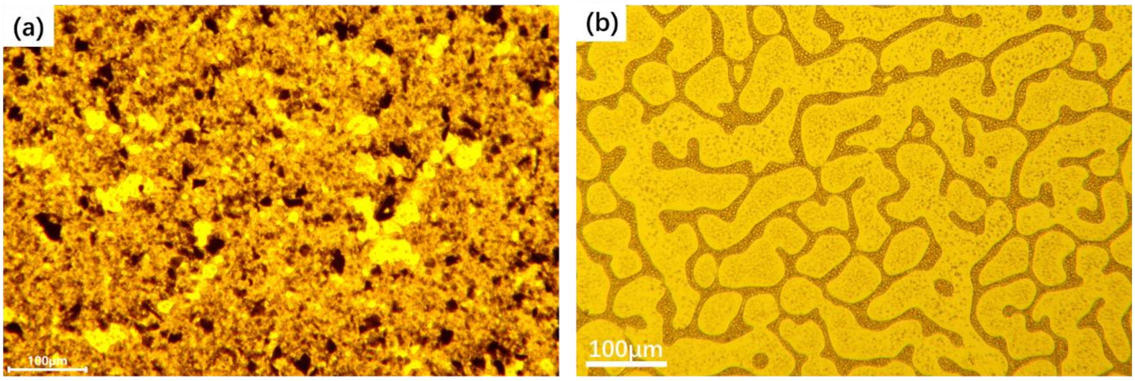

Figure S4. The OM micrographs of (a) DP25R3 and (b) DP25. The dark yellow domains represent the PEI-rich phase, bright yellow domains represent the DGEBA-rich phases, and black sheets represent RGO.

Table S2. The surface tension parameters (in mJ/m^2) of test liquids and static contact angles for DGEBA, PEI, and RGO.

Liquid	γ_L (mJ/m^2)	γ_L^d (mJ/m^2)	γ_L^P (mJ/m^2)	Contact angle (θ)		
				DGEBA	PEI	RGO
Deionized water (DI)	72.8	21.8	51	84.9	82.9	81.2

Glycerol (GL)	64.0	34.0	30	80.4	78.8	65.1
Formamide (FA)	58.0	39.0	19	60.4	62.2	49.7

Table S3. The surface tension parameters (in mJ/m^2) for the free surfaces of DGEBA, PEI, and RGO.

Materials	γ* (mJ/m^2)	γ^d (mJ/m^2)	γ^P (mJ/m^2)
DGEBA	44.23	31.75	12.48
PEI	32.24	26.74	5.50
RGO	38.93	34.86	4.07

$*\gamma = \gamma^d + \gamma^P$

Table S4. Comparison of electrical properties of previously published filler ternary polyblend systems with those of the systems in the current work.

Samples	Polymer A	Polymer B	Filler Loading (wt. %)	Volume Resistivity (Ω cm)	Volume Conductivity (S/m)	Ref
EP/PEI/GnPs	DGEBA	PEI	2	5.7×10^5	—	[1]
PUF@GF/Epoxy	DGEBA	PU foam	8.04 vol%	—	10^{-9}	[2]
MWCNT+GNP/Epoxy	DGEBA	—	2+3	3.1×10^{-3}	3.1×10^{-3}	[3]
EP/35PEI/GnPs	DGEBA	PEI	2	$\approx10^7$	$\approx10^{-5}$	[4]
EP/PEI/CB	DGEBA	PEI	1	$\approx10^3$	—	[5]
DGEBA/PEI/MWCNTs	DGEBA	PEI	2	3.86×10^6	—	[6]
DGEBA/PEI/AgNWs	DGEBA	PEI	3	9.6×10^5 Ω (surface R)	—	[7]
DGEBA/PEI/RGO	DGEBA	PEI	1	2.68	37.31	This work
			2	0.82	121.21	
			3	0.27	366.3	

GF: graphene fluoride, GnPs: graphene nanoplatelets, CB: carbon black, MWCNTs: multiwall carbon nanotubes.

Table S5. The TGA spectra of samples under N_2 atmosphere.

Sample	Temperature range (°C)	Assignment	Residues at 800 °C (wt.%)	Specific RGO loading in the composite (wt.%)
GO	＜100	(~16.9 %) The loss of physisorbed water molecules	19.9	___
	145-285	(~31.5 %) The decomposition of oxygen-containing functional groups		
	285-645	(~15 %) The pyrolysis of less stable (CO, CO_2)		
RGO	＜145	(~2.2 %) The release of water molecules	69.5	___
	145-285	(~9.3 %) The decomposition of oxygen-containing functional groups		

	285-645	(~13.4 %) The pyrolysis of less stable (CO, CO_2)		
Neat DGEBA	200-475	(~90.6 %) The decomposition of DGEBA	2.9	___
	475-675	(~6.5%) The decomposition of few DGEBA		
DR3	200-475	(~90.6 %) The decomposition of DGEBA	5.5	2.60
	475-675	(~3.9%) The decomposition of few DGEBA		
DP5R3	200-475	(~86.3 %) The decomposition of DGEBA and non-aromatic part of PEI	8.3	2.63
	475-675	(~5.4 %) The decomposition of aromatic part of PEI		
DP25R3	200-475	(~76.8 %) The decomposition of DGEBA and non-aromatic part of PEI	11.0	2.83
	475-675	(~12.2 %) The decomposition of aromatic part of PEI		
DP30R3	200-475	(~77.9 %) The decomposition of DGEBA and non-aromatic part of PEI	10.1	2.80
	475-675	(~12 %) The decomposition of aromatic part of PEI		

Table S6. The glass transition temperature T_g (taken as the intersection of the extrapolation of baseline with the extrapolation of the inflexion) of samples.

**Materials**	**Glass transition temperature T_g (°C)**
DR1	94.0
DR1.5	95.1
DR2	95.6
DR2.5	96.7
DR3	97.2
DP30R1	95.7
DP30R1.5	97.3
DP30R2	103.6
DP30R2.5	105.1
DP30R3	110.9

Table S7. The storage modulus and T_g of various samples.

**Samples**	**Glassy modulus E' (MPa)**	**Rubbery modulus (MPa)**	**Glass transition temperature (°C)**	
			T_{g1}	T_{g2}
Neat DGEBA	2532	11	116.1	
DR3	2650	12	117.1	
DP25	2666	79	125.6	193.6
DP25R3	2686	37	127.5	190.1
DP30	2687	75	121.1	186.5

DP30R3	2917	136	126.2	201.2

T_g of neat PEI: 217℃

Table S8. Comparison of EMI shielding performance with other composites.

Samples	Filler content (wt.%)	Thickness (mm)	Frequency (GHz)	EMI SE (dB)	Ref
PS/EMA/VCB	10	1	8.2-12.4	20.1	[8]
Epoxy/RGO	15	2	8.2-12.4	21	[9]
Epoxy/MWCNT	3	2.8	13-18	7.1	[10]
Epoxy/RGO/Fe_3O_4	8.97	2	8.2-12.4	13.5	[11]
PEI/graphene	10	2.3	8.2-12.4	13	[12]
PMMA/Fe_3O_4@MWCNTs	7	2.5	8-12	13.1	[13]
PEI/Graphene@Fe_3O_4	10	2.5	8-12	17	[14]
EP/GNPs/rGO	7.9	3	8.2-12.4	~25	[15]
epoxy/rGO/carbonyl iron	—	3	8.2-12.4	~20	[16]
DGEBA/PEI/RGO	3	2	8.2-12.4	25.9	This work

References

1. Pan, Y.-L.; Ma, C.-G.; Wan, H.-M.; Tao, P.-Y.; Shi, Q.; Huang, D.-S.; Wang, J.-X. Effect of Graphene Nanoplates on Phase Structure and Electrical Properties of Epoxy/Polyetherimide Composite. **2016**.
2. Mani, D.; Vu, M.C.; Jeong, T.-H.; Kim, J.-B.; Lim, C.-S.; Lim, J.-H.; Kim, K.-M.; Kim, S.-R. 3D structured graphene fluoride-based epoxy composites with high thermal conductivity and electrical insulation. *Composites Part A: Applied Science and Manufacturing* **2021**, *149*, 106585.
3. Rajan, J.S. Effective use of nano-carbons in controlling the electrical conductivity of epoxy composites. *Composites Science and Technology* **2021**, *202*, 108554.
4. Wan, H.-M.; Ma, C.-G.; Xi, D.-Y.; Pan, Y.-L.; Shi, Q.; Tao, P.-Y. Effect of Carbon-based Nanofillers on Electrical Properties of Epoxy/Polyetherimide Composites. In *ADVANCED MATERIAL SCIENCE AND ENGINEERING AMSE2016*; World Scientific: 2016; pp. 19-24.
5. Ma, C.G.; Xi, D.Y.; Liu, M. Epoxy resin/polyetherimide/carbon black conductive polymer composites with a double percolation structure by reaction-induced phase separation. *Journal of Composite Materials* **2013**, *47*, 1153-1160.
6. Wang, X.; Li, W.; Zhang, Z.; Chen, K.; Gan, W. Selective localization of multi-walled carbon nanotubes in epoxy/polyetherimide system and properties of the conductive composites. *Journal of Applied Polymer Science* **2019**, *136*, 47911.
7. Liu, Y.; Yang, X.; Yue, L.; Li, W.; Gan, W.; Chen, K. Selective dispersion of silver nanowires in epoxy/polyetherimide binary composites with enhanced electrical conductivity: a study of curing kinetics and morphology. *Polymer Composites* **2019**, *40*, 4390-4401.
8. Ghosh, S.K.; Das, T.K.; Ghosh, S.; Remanan, S.; Nath, K.; Das, P.; Das, N.C. Selective distribution of conductive carbonaceous inclusion in thermoplastic elastomer: A wet chemical approach of promoting dual percolation and inhibiting radiation pollution in

X-band. *Composites Science and Technology* **2021**, *210*, 108800.

9. Liang, J.; Wang, Y.; Huang, Y.; Ma, Y.; Liu, Z.; Cai, J.; Zhang, C.; Gao, H.; Chen, Y. Electromagnetic interference shielding of graphene/epoxy composites. *Carbon* **2009**, *47*, 922-925.
10. Li, J.; Zhang, G.; Ma, Z.; Fan, X.; Fan, X.; Qin, J.; Shi, X. Morphologies and electromagnetic interference shielding performances of microcellular epoxy/multi-wall carbon nanotube nanocomposite foams. *Composites Science and Technology* **2016**, *129*, 70-78.
11. Liu, Y.; Lu, M.; Wu, K.; Yao, S.; Du, X.; Chen, G.; Zhang, Q.; Liang, L.; Lu, M. Anisotropic thermal conductivity and electromagnetic interference shielding of epoxy nanocomposites based on magnetic driving reduced graphene oxide@ Fe3O4. *Composites Science and Technology* **2019**, *174*, 1-10.
12. Ling, J.; Zhai, W.; Feng, W.; Shen, B.; Zhang, J.; Zheng, W.G. Facile preparation of lightweight microcellular polyetherimide/graphene composite foams for electromagnetic interference shielding. *ACS applied materials & interfaces* **2013**, *5*, 2677-2684.
13. Zhang, H.; Zhang, G.; Li, J.; Fan, X.; Jing, Z.; Li, J.; Shi, X. Lightweight, multifunctional microcellular PMMA/Fe_3O_4@ MWCNTs nanocomposite foams with efficient electromagnetic interference shielding. *Composites Part A: Applied Science and Manufacturing* **2017**, *100*, 128-138.
14. Shen, B.; Zhai, W.; Tao, M.; Ling, J.; Zheng, W. Lightweight, multifunctional polyetherimide/graphene@ Fe3O4 composite foams for shielding of electromagnetic pollution. *ACS applied materials & interfaces* **2013**, *5*, 11383-11391.
15. Liang, C.; Qiu, H.; Han, Y.; Gu, H.; Song, P.; Wang, L.; Kong, J.; Cao, D.; Gu, J. Superior electromagnetic interference shielding 3D graphene nanoplatelets/reduced graphene oxide foam/epoxy nanocomposites with high thermal conductivity. *Journal of Materials Chemistry C* **2019**, *7*, 2725-2733.
16. Chen, Y.; Zhang, H.-B.; Huang, Y.; Jiang, Y.; Zheng, W.-G.; Yu, Z.-Z. Magnetic and electrically conductive epoxy/graphene/carbonyl iron nanocomposites for efficient electromagnetic interference shielding. *Composites Science and Technology* **2015**, *118*, 178-185.
